# Health care needs of elderly patients with lung, liver, or colon cancer in Taiwan

**DOI:** 10.1186/s12904-021-00708-3

**Published:** 2021-01-23

**Authors:** Tzu-Yin Lee, Henny Dwi Susanti, Kuo-Chen Hung, Su-Yueh Yang, Hui-Fen Fang, Jia-Ruey Tsai, Jeng-Fong Chiou, Min-Huey Chung

**Affiliations:** 1 School of Nursing, College of Nursing, Taipei Medical University, No. 250, Wu-Xing Street, Taipei, Taiwan; 2 Department of Nursing, Faculty of Health Science, University of Muhammadiyah Malang, Malang, East Java Indonesia; 3 Department of Computer Science and Information Management, Hungkuang University, Taichung City, Taiwan; 4 Department of Nursing, Wan Fang Hospital, Taipei Medical University, Taipei, Taiwan; 5 Center for Nursing and Healthcare Research in Clinical Practice Application, Wan Fang Hospital, Taipei Medical University, Taipei, Taiwan; 6 Department of Nursing, Taipei Cancer Center, Taipei Medical University, Taipei, Taiwan; 7 Cancer Center, Taipei Medical University Hospital, Taipei, Taiwan; 8 Nursing Services, Taipei Medical University Hospital, Taipei, Taiwan; 9 Division of Hematology and Oncology, Department of Internal Medicine, Taipei Medical University Hospital, Taipei Medical University, Taipei, Taiwan; 10 Taipei Cancer Center, Taipei Medical University, Taipei, Taiwan; 11 School of Medicine, College of Medicine, Taipei Medical University, Taipei, Taiwan; 12 Department of Nursing, Shuang-Ho Hospital, Taipei Medical University, New Taipei City, Taiwan

**Keywords:** Cancer, Elderly population, Health care needs, Supportive care needs survey

## Abstract

**Background:**

Globally, different age groups in the elderly population have experienced major shifts over time. Human life expectancy doubled from the 19th to the twentieth century and has increased to 80 years in the twenty-first century. These conditions imply economic challenges and the increasing prevalence of certain health conditions. Old age is associated with increased care needs in various aspects of daily life. This study assessed the health care needs of elderly patients with lung, liver, and colorectal cancer in Taiwan and analyzed the factors underlying their needs.

**Methods:**

This cross-sectional descriptive survey assessed 234 elderly patients with diagnosis of lung, liver, and colorectal cancer in Taiwan. We investigated their health care needs and daily living functions by using the Supportive Care Needs Survey and Karnofsky Performance Status, respectively.

**Results:**

Patients required the most assistance in physical functioning and daily living. Patients aged ≥85 years required more care than those aged 65–74 years in terms of information access and sexuality needs. Patients with poor functional status required more care than those capable of undertaking normal activities. Patients diagnosed as having liver cancer required more care than those with lung or colorectal cancer. Patients with advanced cancer required more physical and daily care than those with early-stage cancer.

**Conclusions:**

Patients’ health care needs differed with age, primary cancer site, and functional status. Patients aged ≥85 years and those with poor function, primary liver cancer, and advanced cancer had higher care needs.

## Background

The World Health Organization (WHO) reported that the cancer burden had globally increased to 18.1 million, of which 9.6 million people died from cancer in 2018 [1]. Cancer is a leading cause of death in 28 countries. The highest death rates occur in North America, Northern and Western Europe, and especially in the Netherlands, Denmark, China, Australia, New Zealand, and Hungary [1]. Similarly, cancer is a main cause of death in Taiwan [2]. According to 2019 Taiwan Health and Welfare Report, cancer has been the major cause of death in Taiwan for over 30 years, and lung, liver and colorectal cancer are the three most common cause of cancer death [3, 4]. In 2019, malignant tumors accounted for 24.9% of deaths among individuals older than 65 years [2]. With Taiwan’s ageing population and growing cancer prevalence, the health care needs of elderly individuals with cancer requires further investigation.

Over the past few decades, researchers have extensively focused on cancer treatment. With advanced medical technology, the care needs of cancer patients have gained more attention. Health care needs, such as health education, disease prevention, diagnosis, treatment, and hospice care, are the needs that can benefit from health care services [5]. Hoekstra, Heins and Korevaar [6] divided the care needs of patients with cancer into four domains: medical, informational, psychological, and proactive contact. Health care needs differ from patients to patients. For example, patients may express more psychological concerns at the time of diagnosis than they would during treatment. Individual differences in age, country, region, marital status, education, occupation, and others can also considerably affect the care needs of these patients [7, 8].

Most cancer studies have focused on supportive care needs related to cancer site, treatment type, or time since diagnosis [9–11] Furthermore, elderly patients have not been specifically analyzed as most studies targeted adult patients [12, 13]. But the care needs of older people, especially needs for physical care and pain elimination, are more complex than those of other age groups [14, 15]. In addition, studies have found that those with lung cancer have higher supportive care needs than other patients [16–21]. However, limited information is available on how to meet the supportive care needs of patients with lung, liver, or colon cancer. Studies have focused only on patients with lung cancer [3, 9, 18] and not on those with liver or colon cancers. Thus, further investigations are required for supportive care in Taiwan’s lung, liver, or colon cancer patients. The present study specifically assessed these patients’ health care needs and analyzed their underlying factors, including age, cancer site, cancer stage, and functional status.

## Methods

### Study design and participants

This study was a multicenter cross-sectional descriptive survey. The sample was selected using a two-stage stratified cluster sampling method. Residential areas including Northern, Central, Southern, and Eastern Taiwan and its outlying islands were the sampling units in the first stage. Hospitals (ten in total) were the sampling units in the second stage. The inclusion criteria were as follows: (1) age ≥ 65 years; (2) physician diagnosis of lung, bronchus, or tracheal cancer; liver or intrahepatic bile duct cancer; or colorectal cancer, which are the three most common cancer types causing mortality in Taiwan [4]; (3) conscious and able to communicate in Chinese or Taiwanese; and (4) no mental impairment such as dementia. The required sample size was estimated using G*power 3.1 [22]. The effect size was set to 0.15 with an alpha value of 0.05. The number of predictors was set to 10, and the power was set to 0.95. The estimated sample size for linear multiple regression was 172, allowing for 20% missing data. This study required at least 206 participants. Therefore, in this study, we enrolled 234 participants and distributed 247 questionnaires. Thirteen questionnaires were excluded due to missing data, yielding a completion rate of 94.7%. The 10 predictors used in this study were sex, age, residential area, education, marital status, chronic disease, Karnofsky Performance Status (KPS), primary cancer site, cancer stage, and treatment.

### Instruments

#### Participant characteristics

A self-reported questionnaire was designed to collect the demographic data of age, sex, area of residence, education level, and marital status. Patients also reported their disease-related characteristics, including chronic diseases (cardiovascular diseases; digestive diseases; respiratory diseases; diabetes; hypertension; urinary system diseases; musculoskeletal disorders; stroke; and ear, nose, and throat disorders), primary cancer site, cancer stage, and treatment received.

#### Supportive care needs survey

The Supportive Care Needs Survey (SCNS) is used to evaluate the perceived needs of people diagnosed with cancer; it consists of 59 items and was originally developed by Bonevski and Sanson-Fisher [23]. Boyes and Girgis [24] developed a shorter version of the SCNS consisting of 34 items (hereafter SCNS-SF34) and five subscales, namely physical and daily living needs, psychological needs, patient care and support needs, health system and informational needs, and sexuality needs [23]. It is a self-reported questionnaire that measures the unmet needs of patients with cancer within 1 month after diagnosis. The tool is measured on a 5-point scale ranging from 1 (no need) to 5 (high need for help), with higher scores reflecting higher needs. The scores for each domain range between 0 and 100, and the standard score for each domain should be 50 [25]. The five domains in this instrument exhibit high internal consistency with Cronbach’s alpha coefficients of 0.86 to 0.96, and they exhibit convergent validity with three other psychosocial measures of well-being [24]. We translated the SCNS-SF34 into Chinese and invited five nursing experts to review the Chinese version for content validity. In our study, the Cronbach’s alpha values of the five domains ranged from 0.78 to 0.95.

#### Functional status

KPS was used to assess patients’ general function and activities of daily living [26] on a scale of 0 to 100, where 100 represents perfect health and 0 represents death. The interrater reliability of the scale is 0.97, and scores are strongly related to other independent measures of patient function [27]. In this study, on the basis of their KPS scores, participants’ functional status was classified into three categories: normal activity, inability to work, and inability to care for oneself [28].

The first category is normal activity (KPS score: 80–100); this means that the patient is able to engage in normal activities and work and requires no special care. The second category is inability to work (KPS score: 50–70); this means that patients are able to live at home and care for most of their own personal needs, with varying levels of assistance required. The last category is inability to care for oneself, and the functional status can be classified into five groups based on the following KPS scores: 40 denotes disability, 30 denotes severe disability, 20 denotes severe illness, 10 denotes moribundity, and 0 denotes death [26]. A functional status value of 80 means that the patient can engage in normal activities.

### Data collection

From January to September 2017, the researchers recruited appropriate candidates from the cancer wards or outpatient clinics of 10 hospitals (4 medical centers, 5 regional hospitals, and 1 district hospital) in Taiwan. Patients completed the self-reported anonymous questionnaire after providing informed consent. The functional status of patients was evaluated by the nurses or researchers. If patients experienced difficulty reading or writing, the nurses or researchers provided assistance.

### Statistical analysis

Data were analyzed using SPSS version 19.0 for Windows (SPSS, Inc., Chicago, IL, USA). Participants were categorized into three groups: young-old (age = 65–74 years), old-old (age = 75–84 years), and oldest-old (age ≥ 85 years) in accordance with other studies of elderly patients in Taiwan [29, 30]. To provide missing data from the SCNS-SF34, we input the mean value of other items in the domain [25]. The Likert summated score for each domain of care needs was standardized on a scale of 0 to 100 as follows [25]: If *m* is the number of questions in the subscale and *k* is the maximum response value for each item, the standardized score is obtained by summing each item, subtracting *m*, and multiplying the result by 100/[*m* × (*k* − 1)]. Patients’ demographic data were described using a frequency assignment table, and the data were expressed as average and standard deviation (SD). The univariate and hierarchical linear regression analyses were performed to determine the significant predictors of total care needs. A stepwise regression model was used to compare the levels of need among various groups stratified by age, education, cancer site, and functional status. All statistical tests were two-tailed, and *p* < 0.05 was considered statistically significant.

## Results

### Patient characteristics

We included 234 elderly patients with lung, liver, and colorectal cancer (Table 1). The study sample comprised 103 women (44.0%) and 131 men (56.0%). The young-old, old-old, and oldest-old groups consisted of 150 (64.1%), 68 (29.1), and 16 (6.8%) participants, respectively. The mean age was 73.3 with a standard deviation (SD) of 6.3 years. Participants’ areas of residence were distributed in Northern (21.4%), Central (24.8%), and Southern (42.3%) Taiwan and the Eastern region and outlying islands (11.5%) of Taiwan. Approximately 17.1% of patients had no formal education, 43.2% had a primary education, and 39.7% had a junior high school education or higher. Most participants were married (68.4%) and had at least one chronic disease (68.4%). The liver and intrahepatic bile duct were the primary sites of cancer in 64 respondents (27.4%); the lung, bronchus, and trachea were the primary sites in 70 respondents (29.9%); and the colon, rectum, and anus were the primary sites for 100 respondents (42.7%). Overall, 22.2% of participants had cancer in stages 0–II; 20.1% had stage III; 29.5% had stage IV; and the remaining 28.2% did not know their cancer stage. Participants may receive more than one cancer treatments. 61.1% of them reported having received chemotherapy, 17.9% reported having received surgery, 16.2% reported having received radiotherapy, and 22.6% reported having received other type(s) of cancer treatments. 84.6% of the patients received only one type of cancer treatment. 15.4% of the patients received more than two types of cancer treatments.

**Table 1:** Demographics and Disease Characteristics of All Participants (*n* = 234)

Variable	N	%
**Sex**		
Female	103	44.0
Male	131	56.0
**Age (year)**		
65–74	150	64.1
75–84	68	29.1
≥ 85	16	6.8
**Residential area**		
Northern	50	21.4
Central	58	24.8
Southern	99	42.3
Eastern and Islands	27	11.5
**Education**		
No formal education	40	17.1
Primary school	101	43.2
Junior high school or above	93	39.7
**Marital status**		
Unmarried	74	31.6
Married	160	68.4
**Chronic diseases**		
No	74	31.6
Yes	160	68.4
**Primary cancer site**		
Liver or intrahepatic bile duct	64	27.4
Lung, bronchus, or trachea	70	29.9
Colon, rectum, or anus	100	42.7
**Cancer stage**		
Unknown	66	28.2
0–II	52	22.2
III	47	20.1
IV	69	29.5
**Treatment**^**a**^		
Surgery	42	17.9
Chemotherapy	143	61.1
Radiotherapy	38	16.2
Other	53	22.6
Variable	Mean	SD
**Age**	73.3	6.3
**Karnofsky performance status**	75.0	19.8
**Total supportive care needs**	79.7	20.5
Physical and daily living needs^b^	38.5	22.5
Psychological needs^b^	35.3	19.4
Patient care and support needs^b^	26.7	15.1
Health system and information needs^b^	33.1	18.9
Sexuality needs related to sexual relationships^b^	33.3	21.4

^a^Percentages are proportions of total; respondents may indicate more than one category

^b^Score for each domain of care needs calculated using standardized methods

The mean KPS score was 75.0 (SD: 19.8). The mean score for all patients’ total supportive care needs was 79.7 (SD: 20.5). After standardization, participants’ mean scores were 38.5 (SD: 22.5) for physical and daily living needs, 35.3 (SD: 19.4) for psychological needs, 26.7 (SD: 15.1) for patient care and support needs, 33.1 (SD: 18.9) for health system and information needs, and 33.3 (SD: 21.4) for sexuality needs.

### Care needs in the SCNS

Univariate linear regression models were used to investigate the variables potentially influencing total care needs. As presented in Table 2, age was significantly correlated with supportive care needs, and the oldest-old group had a higher mean level of such needs than the young-old group did. The mean level of these needs for the oldest-old group was 91.3 (SD: 24.3), which was higher than that for the young-old group. Education was significantly associated with the level of supportive care needs. Patients with primary school, junior high school, and higher levels of education required less care than did those with no formal education. Patients diagnosed as having primary liver cancer required more care than those with primary lung or colorectal cancer. Participants receiving radiotherapy required a significantly higher level of care (*p* < 0.001) than did those not receiving radiotherapy. The higher the KPS score, the less care was required (*p* < 0.001).

**Table 2:** Analysis for Total Supportive Care Needs

Variable	Mean ± SD	*p*
**Sex**		0.06
Female	82.6 ± 21.6	
Male	77.5 ± 19.4	
**Age**		0.04*
65–74	77.9 ± 19.8	
75–84	81.0 ± 20.4	
≥ 85	91.3 ± 24.3	
**Residential area**		0.34
Northern	79.9 ± 19.4	
Central	76.6 ± 16.0	
Southern	82.2 ± 22.6	
Eastern and Islands	76.9 ± 22.7	
**Education**		0.03*
No formal education	87.3 ± 24.8	
Primary school	77.2 ± 19.4	
Junior high school or above	79.2 ± 19.1	
**Marital status**		0.39
Unmarried	81.4 ± 19.4	
Married	78.9 ± 21.0	
**Chronic diseases**		0.33
No	77.8 ± 20.4	
Yes	80.6 ± 20.6	
**Karnofsky performance status**	79.7 ± 20.5	0.00**
**Primary cancer site**		0.01*
Liver or intrahepatic bile duct	86.6 ± 22.3	
Lung, bronchus, or trachea	76.2 ± 19.3	
Colon, rectum, or anus	77.7 ± 19.3	
**Cancer stage**		0.94
Unknown	79.9 ± 19.2	
0–II	79.6 ± 23.6	
III	78.2 ± 21.3	
IV	80.7 ± 19.0	
**Treatment**		
Surgery (Yes vs. No)	84.2 ± 21.9	0.11
Chemotherapy (Yes vs. No)	80.0 ± 21.1	0.49
Radiotherapy (Yes vs. No)	91.6 ± 23.0	0.00**
Other (Yes vs. No)	79.7 ± 19.1	0.99

Independent t-test and one-way ANOVA for categorical variables; Pearson correlation for continuous variables; *P < 0.05; **P < 0.001

Table 3 presents the results of hierarchical regression models for total care needs. Model 1 included sex, age, area of residence, education level, marital status, chronic disease, and KPS score as independent variables. The significant variables were education level and functional status, with *R*^2^ = 0.19. In model 2, primary cancer site was added to the regression model and significantly affected the level of care needs, with *R*^2^ = 0.21, indicating a change of 0.02 in the *R*^2^ value. We added cancer stage as an independent variable in model 3, and *R*^2^ = 0.21. However, the coefficient was nonsignificant, with a 0.00 increase in *R*^2^. In model 4, we added treatment received as a variable. Radiotherapy treatment significantly increased the level of care needs; *R*^2^ was 0.25, representing an increase of 0.04.

**Table 3:** Hierarchical Regression Model of Care Needs (*n* = 234)

Variable	Model 1	Model 2	Model 3	Model 4
**Sex** (Male vs. Female)	−0.08	−0.10	−0.10	−0.11
**Age**				
65–74	Ref.	Ref.	Ref.	Ref.
75–84	−0.02	−0.02	−0.02	−0.01
≥ 85	0.07	0.07	0.06	0.08
**Residential area**				
Northern	Ref.	Ref.	Ref.	Ref.
Central	−0.02	−0.00	−0.00	0.02
Southern	0.07	0.05	0.05	0.06
Eastern and Islands	−0.00	0.04	0.05	0.05
**Education**				
No formal education	Ref.	Ref.	Ref.	Ref.
Primary school	−0.19*	−0.17	− 0.18	− 0.16
Junior high school or above	−0.12	−0.10	−0.10	−0.08
**Marital status** (Married vs. Unmarried)	0.02	0.01	0.01	−0.01
**Chronic diseases** (Yes vs. No)	0.03	0.02	0.03	0.01
**Karnofsky performance status**	−0.36**	− 0.35**	− 0.37**	− 0.31**
**Primary cancer site**				
Liver or intrahepatic bile duct		Ref.	Ref.	Ref.
Lung, bronchus, or trachea		−0.20*	−0.19*	−0.19*
Colon, rectum, or anus		−0.14	−0.13	−0.14
**Cancer stage**				
Unknown			Ref.	Ref.
0–II			−0.02	−0.02
III			0.00	−0.02
IV			−0.05	−0.04
**Treatment**				
Surgery (Yes vs. No)				0.13
Chemotherapy (Yes vs. No)				0.14
Radiotherapy (Yes vs. No)				0.20*
Others (Yes vs. No)				0.09
R^2^	0.19	0.21	0.21	0.25
R^2^ change	0.18	0.02	0.00	0.04

*P < 0.05; **P < 0.001

### Five domains of care needs in the SCNS

**Fig. 1:**
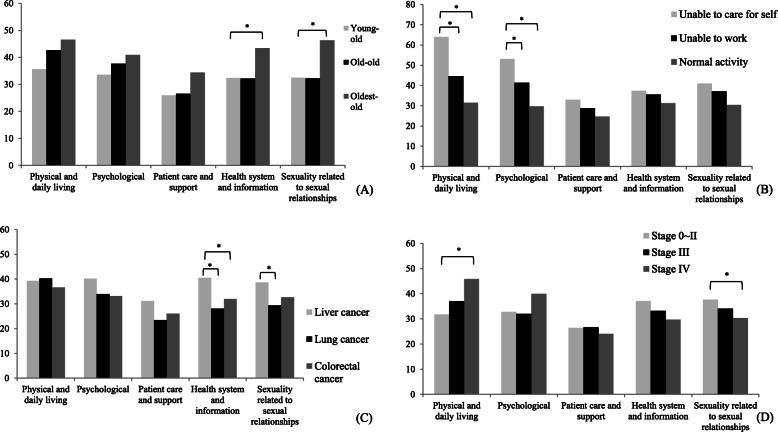
Standardized score of each domain of care needs for various (**a**) age groups, (**b**) Karnofsky Performance Status (KPS) scores, (**c**) primary cancer sites, and (**d**) cancer stages. *Significance was tested using stepwise regression; variables included sex, age, residential area, education, marital status, chronic diseases, primary cancer site, cancer stage, treatment received, and KPS. Patients with unknown cancer stage were excluded from analysis in (**d**). Young-old: age = 65–74 years; old-old: age = 75–84 years; and oldest-old: age ≥ 85 years. Normal activity: score = 80–100; unable to work: score = 50–70; and unable to care for oneself: score = 10–40

The standardized scores for each domain of care needs in the categories of age, functional status, primary cancer site, and cancer stage are presented in Fig. 1. We tested the effects of the aforementioned variables on each domain of care needs by using a stepwise regression model. In the health information and sexuality domain, the oldest-old patients required a significantly higher level of care than did the young-old group. Patients unable to care for themselves required a higher level of both physical daily living and psychological care than did patients who were only unable to work or those engaging in normal activity. Patients with primary lung or colorectal cancer had lower health system and information needs than patients with primary liver cancer did. Furthermore, patients with primary liver cancer had higher sexuality needs than those with lung cancer. Participants with stage-IV cancer required higher levels of physical daily living care than those with cancer stages 0–II did; however, they exhibited a lower level of sexuality needs.

## Discussion

The strength of this study is that it is a nationwide survey to investigate health care needs in elderly patients with lung, liver, and colorectal cancer. The results provide a reference indicating the medical services that will be required in Taiwan. Policies for improving the health care of cancer patients aged > 85 years are imperative. Sanson-Fisher and Girgis [12] reported that the likelihood of reporting care needs did not differ between individuals aged 18–30 and 61–70 years and those aged 71–90 years. However, the results of the present study in Table 1, which investigated people aged above 65 years old, are similar to those of Ahn and Kim [15], who demonstrated that age is positively correlated with the need for and use of general health care services among elderly patients [15, 31]. In Taiwan, age is a major predictor of health care needs [32]. Among cancer patients, older age indicates poorer health status [33], which may be the reason why the oldest-old patients reported higher health care needs than the other patients did.

This study demonstrated a significant relationship between KPS and supportive care needs (Table 2). This is consistent with the results from previous studies, showing that cancer patients who experience the most serious symptoms resulted in decreased physical function in daily life [34]. Patients with higher functional status have lower care needs, whereas patients who are unable to care for themselves have higher care needs, especially physical and psychological needs, as they experience psychological disorders and physical limitations in fulfilling daily life needs. A Taiwan study indicated that functional disability is a major predictor of care needs among elderly patients [32]. People past middle age and with functional difficulties are significantly more likely to report poor mental health [35]. Thus, caregivers must provide more psychological and spiritual support to patients with poor functional status and must assist with their daily physiological problems.

Compared with patients with primary lung or colorectal cancer, those with primary liver cancer have more health care information and sexuality needs. Information and education are among the key domains of unmet supportive care needs in people with chronic liver disease [36]. Furthermore, health care professionals are the most common source of information, rather than family and friends [37]. Caregivers must address the distinct information needs of various subsets of patients with cancer as the incidence rate of liver cancer has increased over the past 30 years [38]. Providing intensive nursing care can also be useful in helping patients to find information and motivation related to healing the disease [39]. Intensive nursing care includes reporting of symptoms and abnormal patient conditions quickly to prevent complications [40].

Although the present study’s regression model did not find a significant correlation between overall support needs and cancer stage, patients with lung, liver, and colorectal cancer in stage IV exhibited higher physical daily living needs, compared with patients in stages 0–II (Fig. 1d). Another study discovered that patients with cancer in stages III or IV had more unmet supportive care needs than did patients with other cancer stages [41]. A meta-analysis revealed that patients with advanced or terminal-stage illness experienced more severe pain than patients who continued to receive treatment [42]. The frequent pain experienced by these patients may have increased their physical care needs. Thus, health care providers may pay attention to pain management in patients with advanced cancer to reduce excessive medical expenditures.

The present study revealed that patients receiving radiotherapy require more care (Table 3). Other studies have shown that cancer patients who receive radiotherapy treatment have psychological and sexual needs [10, 43]. For patients with primary rectal cancer, preoperative radiotherapy can increase sexual dysfunction and reduce daily activity levels [44]. Cancer patients who receive multiple treatments have more health care needs than those with single treatment because the combination of treatments has more side effects and complications such as hair loss, sleep disturbance, loss of appetite and shortness of breath [45]. Most cancer patients in this study demand more health care needs support than others as they receive not only radiotherapy but also multiple treatments at the same time. Future studies must further investigate the care needs of patients undergoing radiotherapy to provide services that improve their quality of life. Several previous studies reported that providing information to patients and their families about the condition of the disease improves adaptation during treatment and show positive results and reduces disease recurrence [46–48]. Therefore, one of the main roles of nurses is to provide education to patients.

Oldest-old patients had higher overall care needs than young-old patients, especially in health system information needs and sexuality needs (Fig. 1a). Aging is related to a higher risk of health problems, which necessitate access to information and utilization of health care services [49]. With the ageing global population, health-related information is required to address the health conditions of elderly people [50], as oldest-old patients could not take care of themselves and had additional psychological and physical needs related to daily living, including limited functional ability, limited ability to walk, the presence of over one disease, and living alone [15]. Therefore, they require total supportive care [51, 52]. Regarding sexuality needs, oldest-old patients had higher sexuality needs compared with other two groups (Fig. 1a). A longitudinal study conducted at Duke University reported that 15% of older people were engaged in sexual activity [53]. However, aging not only causes physical function degradation [54] but also affect psychological factors, such as dissatisfaction of body images, or low self-esteem, which may indirectly influence sexual function [55]. Other authors have explained that lower level of sexual needs reflects the lack of attention from a partner or family toward patients during treatment [56]. As a result, oldest-old patients had higher sexuality need compared with other two groups.

This study followed appropriate procedures and is a nationwide survey of the care needs of elderly patients with lung, liver, and colorectal cancer in Taiwan. Nevertheless, the following limitations should be considered. First, the caregivers of participants—for example, their family members or primary nurses—may have significantly influenced participants’ survey responses, and this potential influence was not analyzed for any correlations with the variables of this study. Second, only patients with lung, bronchus, tracheal, liver, intrahepatic bile duct, or colorectal cancer were included in this research. The care needs of elderly patients with other primary cancers may differ from those of the study group. Third, the study was cross-sectional in nature. We did not consider the care needs of patients at different time points.

## Conclusions

Most of the care needs of elderly patients with lung, liver, and colorectal cancer were in the physical and daily living domains. The health care needs of elderly patients with these kinds of cancers differed according to age, primary cancer site, cancer stage, and general function in daily life. Therefore, appropriate measures should be used for elderly patients with these kinds of cancers, especially the oldest-old and those with poor function, primary liver cancer, and advanced cancer.

This study provided a vision and direction for health administrators; for example, care- and treatment-related decisions should be made with elderly patients and their families through collaboration and discussion. This quantitative study examined the information access and sexuality needs of the oldest-old patients with lung, liver, and colorectal cancer. To the best of our knowledge, the present research was the first to call attention to the problems associated with extreme population aging foreseen in Taiwan. Appropriate measures should be implemented for elderly patients with lung, liver, and colorectal cancer whose care needs are distinct from those of younger patients with these kinds of cancers, and their health care needs should be met as fully as possible. Given that health care needs differ by age, primary cancer site, and functional status, guidelines should be established to determine standard operation processes for the care provided to elderly patients with lung, liver, and colorectal cancer in Taiwan.

## Data Availability

The datasets used and analyzed in the current study are available from the corresponding author on reasonable request.

## Abbreviations

SCNS: Supportive Care Needs Survey
SCNS-SF34: Supportive Care Needs Survey Short Form 34
KPS: Karnofsky Performance Status
WHO: World Health Organization
